# Improvements over time for patients following liver trauma: A 17-year observational study

**DOI:** 10.3389/fsurg.2023.1124682

**Published:** 2023-02-24

**Authors:** Adam Brooks, Danielle Joyce, Angelo La Valle, John-Joe Reilly, Lauren Blackburn, Samuel Kitchen, Louise Morris, David N Naumann

**Affiliations:** ^1^ Major Trauma Department, East Midlands Major Trauma Centre, Queens Medical Centre, Nottingham, United Kingdom; ^2^ Institute of Inflammation and Ageing, University of Aberdeen, Aberdeen, United Kingdom; ^3^ Department of Trauma and Emergency General Surgery, University of Birmingham, Birmingham, United Kingdom; ^4^ University Hospitals Birmingham NHS Foundation Trust, Birmingham, United Kingdom

**Keywords:** liver, hepatic, trauma, injury, hepatobilary injury

## Abstract

**Background:**

Centralisation of trauma care has been shown to be associated with improved patient outcomes. The establishment of Major Trauma Centres (MTC) and networks in England in 2012 allowed for centralisation of trauma services and specialties including hepatobiliary surgery. We aimed to investigate the outcomes for patients with hepatic injury over the last 17 years at a large MTC in England in relation to the MTC status of the centre.

**Methods:**

All patients who sustained liver trauma between 2005 and 2022 were identified using the Trauma Audit and Research Network database for a single MTC in the East Midlands. Mortality and complications were compared between patients before and after establishment of MTC status. Multivariable logistic regression models were used to determine the odds ratio (OR) and 95% confidence interval (95% CI) for complications according to MTC status, accounting for the potentially confounding variables of age, sex, severity of injuries and comorbidities for all patients, and the subgroup with severe liver trauma (AAST Grade IV and V).

**Results:**

There were 600 patients; the median age was 33 (IQR 22–52) years and 406/600 (68%) were male. There were no significant differences in 90-day mortality or length of stay between the pre- and post-MTC patients. Multivariable logistic regression models showed both lower overall complications [OR 0.24 (95% CI 0.14, 0.39); *p *< 0.001] and lower liver-specific complications [OR 0.21 (95% CI 0.11, 0.39); *p *< 0.001] in the post-MTC period. This was also the case in the severe liver injury subgroup (*p *= 0.008 and *p *= 0.002 respectively).

**Conclusions:**

Outcomes for liver trauma were superior in the post-MTC period even when adjusted for patient and injury characteristics. This was the case even though patients in this period were older with more comorbidities. These data support the centralisation of trauma services for those with liver injuries.

## 1. Introduction

The liver is the most common solid organ to be injured following trauma and is the primary cause of death in severe abdominal trauma (1). There has been an evolution in management of liver trauma over the last several decades, with a large body of evidence to suggest that non-operative management (NOM) is safe, even in high grade injuries (2–4).

The Major Trauma Centres (MTC) and networks were initially established in England in 2012, leading to an increase in volume of trauma admissions at each MTC (5). There is some evidence that centralisation of trauma services at MTCs improves outcomes for injured patients (6–8). The combination of key trauma services and specialties at MTCs allows for rapid assessment and management of injured patients (9). It is likely that outcomes following liver trauma in the UK have improved since instigation of the MTCs and the development of the UK trauma networks, but there are limited data regarding this particular group of patients.

The current study aimed to investigate patients who were admitted with liver trauma over the last 17 years at a large MTC in the UK and determine whether there were any differences in both patient and injury characteristics and outcomes after the establishment of MTC status.

## 2. Methods

### 2.1. Study design and setting

An observational study was undertaken to investigate patient outcomes following liver trauma over a 17 year period (2005–2022) at a large Major Trauma Centre in the East Midlands, UK. Institutional approval was granted prior to data collection. The study is reported according to the STROBE guidelines (10).

### 2.2. Patient selection

Patients were included if they were admitted following trauma, with a recorded injury to the liver. All ages of patients were included. Patients were not included if they were discharged directly home from the Emergency Department (ED).

### 2.3. Data collection

Data collected included demographic details [age, gender, comorbidities and Charlson Comorbidity Index (CCI)], injury details (injury severity score (ISS), American Association of Surgery for Trauma (AAST) grade of liver injury and mechanism of injury (blunt or penetrating)). Physiological parameters were recorded for the prehospital period and on admission to ED, and included heart rate, Glasgow Coma Scale (GCS) and systolic blood pressure (SBP). Patient management was recorded, including operative and non-operative.

### 2.4. Definitions

Patients were divided into pre- and post-MTC groups depending on their date of admission, with April 2012 being the start of the post-MTC period. High grade liver trauma was defined as an AAST grade of IV or V. A complication was defined according to the Adapted Clavien-Dindo in Trauma (ACDiT) scale (11) as any complication that required deviation from the initial management plan, and included all patients whether managed surgically or non-operatively. Complications were defined as relating to the liver by the study authors if they were related to liver function, injury or surgery, such as bile leak or liver abscess, or wound complications following hepatic surgery.

### 2.5. Outcomes

The outcomes of interest included 90 day mortality, any complications (as defined above), liver-related complications and length of stay.

### 2.6. Data analysis

Continuous data were summarised using median and interquartile range (IQR) and categorical data were summarised using number and percentage in parentheses. Pairwise analysis was undertaken using Mann–Whitney *U* tests to compare continuous variables and Fisher's exact test for categorical data. Univariable and multivariable logistic regression models were used to determine the odds ratio (OR) and 95% confidence intervals (95% CI) for operative intervention using *a priori* selected potentially confounding variables (age, sex, ISS, CCI and AAST grade IV/V). A planned subgroup analysis was undertaken for patients with high grade liver trauma (i.e., AAST grade IV or V). A *p*-value of <0.05 was considered statistically significant. Analysis was performed using GraphPad V9.4 (GraphPad Software, LLC) and RStudio V1.4 (R Foundation for Statistical Computing, Vienna, Austria).

## 3. Results

### 3.1. Study patient characteristics

There were 600 included patients, with a median age of 33 (22–52) years old and 406/600 (68%) were male, and 74/600 (12%) were children. Table 1 summarises the demographic, injury and physiological data for all patients, with comparison between the patients before (*n* = 100) and after (*n* = 500) MTC status in 2012. Patients were older in the post-MTC group and had an overall higher burden of comorbidities (higher CCI) (Table 1). There was a higher proportion of patients with blunt injury in the post-MTC period but there were no overall differences in recorded pre-hospital or ED physiology (Table 1).

**Table 1:** Patient characteristics compared between those pre- and post- MTC status in 2012.

Patient characteristic^a^	All Patients (*N* = 600)	Pre-MTC (*n* = 100)	MTC (*n* = 500)	*p*-value
Age, years	33 (22–52)	28 (19–42)	35 (22–53)	0.003^b^
Aged >65	69 (12)	3 (3)	66 (13)	0.002^c^
Male sex	406 (68)	65 (65)	341 (68)	0.559
ISS	22 (13–34)	17 (13–34)	24 (13–34)	0.220
AAST Grade	2 (2–3)	2 (2–3)	2 (2–3)	0.622
High Grade (AAST ≥4)	130 (22)	17 (17)	113 (23)	0.224
CCI	0 (0–1)	0 (0–0)	0 (0–1)	<0.001^b^
Blunt injury	492 (82)	71 (71)	421 (84)	0.003^c^
Prehospital physiology				
* Heart rate, bpm*	95 (77–115)	92 (83–111)	95 (76–115)	0.985
* SBP, mmHg*	121 (102–137)	113 (93–134)	121 (103–138)	0.427
* GCS*	15 (14–15)	15 (12–15)	15 (14–15)	0.491
ED physiology				
* Heart rate, bpm*	94 (77–112)	95 (79–114)	93 (75–112)	0.589
* SBP, mmHg*	121 (105–136)	119 (107–140)	121 (105–136)	0.875
* GCS*	15 (14–15)	15 (14–15)	15 (14–15)	0.612
Management				
* Any operation*	321 (54)	46 (46)	275 (55)	0.101
* Laparotomy*	203 (34)	30 (30)	173 (35)	0.419
* Angioembolisation*	20 (3)	3 (3)	17 (3)	1.00
Outcomes				
* Length of stay, days*	8 (4–17)	8 (4–15)	8 (4–18)	0.898
* Length of stay in ICU*	0 (0–4)	0 (0–5)	0 (0–4)	0.195
* 90 day mortality*	43 (7)	6 (6)	37 (7)	0.832
* Any complication*	128 (21)	41 (41)	87 (17)	<0.001^c^
* Liver complication*	61 (10)	23 (23)	38 (8)	<0.001^c^

^a^ Categorical data are reported as *n* (%), and continuous data are reported as median (interquartile range).

^b^ Statistically significant using Mann-Whitney *U* test.

^c^ Statistically significant using Fisher's exact test.

MTC, Major Trauma Centre; ISS, Injury severity score; CCI, Charlson Comorbidity Index; AAST, American Association for the Surgery of Trauma; bpm, beats per minute; SBP, systolic blood pressure; GCS, Glasgow Coma Scale; ED, Emergency Department; ICU, intensive care unit.

### 3.2. Patient management

321/600 (54%) patients required any type of surgery for their injuries. Of these patients, 203 (34% of the whole study cohort) required an emergency laparotomy. Only 23/203 (11%) of those who had a laparotomy (4% of the study cohort) required specific surgical intervention for their liver injury during their laparotomy. All other liver injuries were managed conservatively. Most non-laparotomy operations were trauma and orthopaedic, neurosurgical or cardiothoracic. There was not a significant difference in proportion of patients requiring surgery, non-operative management or angioembolisation between the pre- and post-MTC periods. Patients were more likely to have surgery in instances of penetrating trauma, high grade liver injuries and lower prehospital SBP, but there was no increase in likelihood of surgery after the establishment of the MTC (Table 2). 129/600 (22%) patients were transferred to the MTC from local Trauma Units (TUs) within the East Midlands region.

**Table 2:** Odds ratio of requiring laparotomy following liver trauma according to patient and injury characteristics.

Characteristic	a. Univariable analysis			b. Multivariable analysis		
	OR	95% CI	*p*-value	OR	95% CI	*p*-value
MTC Status	1.23	0.78, 1.99	0.375	2.42	0.42, 17.0	0.341
Age	0.99	0.98, 0.99	0.017^a^	1.00	0.98, 1.02	0.783
Male sex	1.40	0.97, 2.04	0.076	0.91	0.52, 1.58	0.729
ISS	1.01	1.00, 1.02	0.028^a^	1.02	1.00, 1.04	0.131
CCI	0.85	0.74, 0.96	0.011^a^	0.94	0.71, 1.22	0.629
Penetrating injury	11.7	7.2, 19.8	<0.001^a^	33.1	14.0, 88.4	<0.001^a^
High grade^b^	1.26	1.09, 1.45	0.002^a^	2.33	1.27, 4.26	0.006^a^
Prehospital SBP	0.99	0.98, 0.99	<0.001^a^	0.99	0.98, 1.00	0.004^a^
Prehospital GCS	0.95	0.90, 1.00	0.060	0.95	0.89, 1.03	0.211

^a^ Statistically significant.

^b^ AAST Grade IV or V.

OR, Odds ratio; 95% CI, 95% confidence interval; ISS, Injury severity score; AAST, American Association for the Surgery of Trauma; SBP, systolic blood pressure; GCS, Glasgow Coma Scale; CCI, Charlson Comorbidity Index.

### 3.3. Outcomes

**Figure 1:**
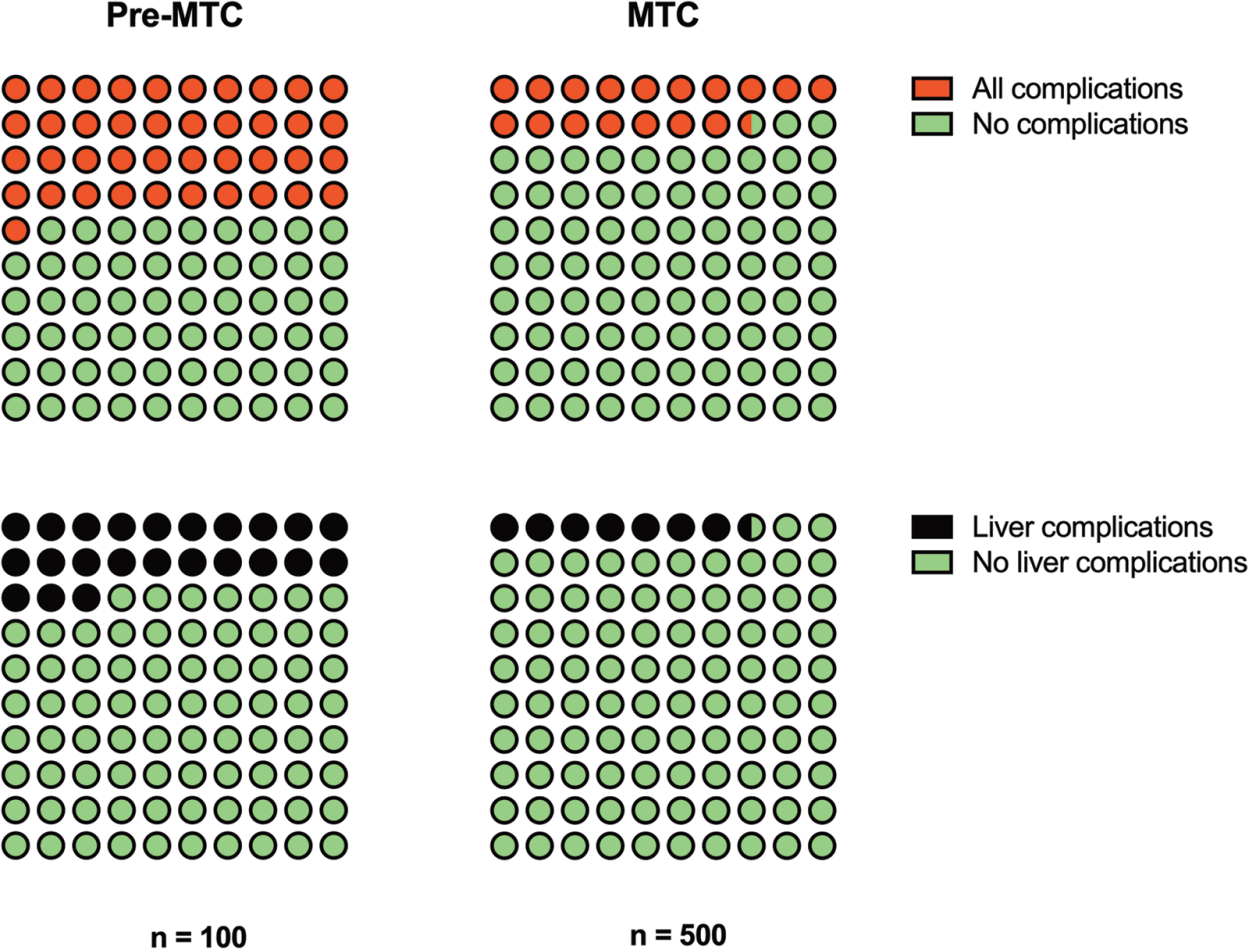
Proportion of patients who had any complications and liver-specific complications compared between the pre-MTC and MTC study periods.

There were no statistically significant differences in length of stay or 90-day mortality between the pre- and post-MTC groups. There were 128/600 (21%) patients who had any complications. There was a large variety in complications including 61 cases of liver complications, 26 cases of pneumonia, 16 neurological complications, 14 superficial surgical site complications, and 10 deep infections. There appeared to be significantly fewer complications (all complications and also liver-specific complications) in the post-MTC period (Table 1 and Figure 1). When all complications and liver-specific complications were examined using logistic regression models that accounted for the potential confounding variables of age, sex, high grade liver injury, CCI and ISS, the MTC time period remained significant (*p *< 0.001 for both all complications and liver-specific complications) (Table 3).

**Table 3:** Odds ratio of having (a) any complications and (b) liver complications according to MTC status adjusted for potentially confounding patient and injury characteristics.

	Characteristic	Univariable analysis			Multivariable analysis		
		OR	95% CI	*p*-value	OR	95% CI	*p*-value
(A) Any complication	MTC status	0.30	0.19, 0.48	<0.001^a^	0.24	0.14, 0.39	<0.001^a^
	Age	1.01	1.00, 1.02	0.104	1.03	1.01, 1.05	0.004^a^
	Male sex	0.89	0.59, 1.35	0.578	1.01	0.65, 1.58	0.974
	AAST grade ≥4	1.20	0.75, 1.89	0.430	1.24	0.74, 2.04	0.402
	CCI	1.00	0.88, 1.13	0.938	0.83	0.65, 1.04	0.126
	ISS	1.03	1.02, 1.04	<0.001^a^	1.03	1.02, 1.05	<0.001^a^
(B) Liver complication	MTC status	0.28	0.16, 0.49	<0.001^a^	0.21	0.11, 0.39	<0.001^a^
	Age	1.00	0.99, 1.02	0.665	1.03	1.00, 1.05	0.043^a^
	Male sex	0.90	0.52, 1.60	0.712	1.05	0.59, 1.95	0.860
	AAST grade ≥4	2.07	1.16, 3.62	0.012^a^	2.22	1.18, 4.11	0.012^a^
	CCI	0.94	0.77, 1.12	0.542	0.78	0.54, 1.10	0.208
	ISS	1.04	1.02, 1.05	<0.001^a^	1.04	1.02, 1.06	<0.001^a^

^a^ Statistically significant.

OR, Odds ratio; 95% CI, 95% confidence interval; ISS, Injury severity score; AAST, American Association for the Surgery of Trauma; CCI, Charlson Comorbidity Index.

### 3.4. Subgroup analysis

There were 130/600 (22%) patients with high grade liver injury including 17 in the pre-MTC period and 113 in the post-MTC period. When the likelihood of complication was analysed in this subgroup, there was again a reduced likelihood of any complications and liver-specific complications in the latter period, even when adjusted for age, sex, comorbidities and ISS (Table 4). Similar to the whole study cohort, there was no significant difference in 90-day mortality (2/17 vs. 8/113 respectively; *p* = 0.619) or length of stay (9 (IQR 6–16) vs. 10 (IQR 5–23) days respectively; *p* = 0.830).

**Table 4:** Odds ratio of having (a) any complications and (b) liver complications according to MTC status adjusted for potentially confounding patient and injury characteristics for patients with high grade liver trauma (AAST grade IV or V).

	Characteristic	Univariable analysis			Multivariable analysis		
		OR	95% CI	*p*-value	OR	95% CI	*p*-value
(A) Any complication	MTC status	0.29	0.10, 0.84	0.021^a^	0.20	0.06, 0.66	0.008^a^
	Age	1.03	1.00, 1.05	0.023^a^	1.03	0.99, 1.08	0.093
	Male sex	0.66	0.29, 1.53	0.328	0.80	0.33, 1.99	0.624
	CCI	1.39	0.95, 2.03	0.080	0.89	0.45, 1.73	0.731
	ISS	1.02	0.99, 1.05	0.140	1.03	1.00, 1.06	0.095
(B) Liver complication	MTC status	0.20	0.07, 0.63	0.005^a^	0.13	0.04, 0.47	0.002^a^
	Age	1.03	1.00, 1.05	0.031^a^	1.03	0.98, 1.08	0.234
	Male sex	1.07	0.41, 3.03	0.893	1.54	0.53, 5.08	0.447
	CCI	1.51	1.00, 2.24	0.040^a^	1.18	0.54, 2.57	0.665
	ISS	1.02	0.99, 1.06	0.133	1.03	1.00, 1.07	0.082

^a^ Statistically significant.

OR, Odds ratio; 95% CI, 95% confidence interval; ISS, Injury severity score; AAST, American Association for the Surgery of Trauma; CCI, Charlson Comorbidity Index.

## 4. Discussion

The main finding from our study of 600 patients with liver trauma was that patients admitted after the MTC designation were less likely to have complications than before (both liver-specific complications and overall complications), even in models that adjusted for age, sex, comorbidities and severity of injury. This was the case for all patients and also the subgroup of AAST Grade IV and V injuries. These findings were present even though patients in the MTC period tended to be older with more comorbidities, when complications might be expected to be naturally higher. Overall mortality was unchanged over the study period.

Other international centres have reported improvements over time in outcomes for patients following liver trauma, including those in Australia (12), New Zealand (13), Korea (14), Norway (15) and South Africa (16). Centralisation of complex specialist surgery is already well established within UK tertiary and quaternary centres for adult and paediatric liver disease, with good evidence of improved outcome, particularly for patients with liver cancers (16) and infants with biliary atresia (17).

Regional centralisation of trauma services has also been demonstrated to result in improved outcomes for all patients admitted with traumatic injuries. The introduction of trauma systems has been associated with reductions in mortality around the world, including in the United States (18), Australia (19) and the Netherlands (20). In England, while unadjusted mortality was not shown to improve following the implementation of the national trauma system in 2012, early analysis of outcomes found a 19% increase in the risk-adjusted survival for trauma patients who reach hospital alive (21). Centralised trauma systems have also been shown to increase efficiency in trauma care, with reductions in time to CT (22, 23) and time to urgent surgery (23) and fewer patients requiring secondary transfers (5). Importantly, more patients are reported to leave hospital with a good functional outcome (20, 22). Each nation and trauma network is unique, and therefore an exploration of the outcomes for patients with liver injuries in our UK trauma network was justified.

As well as centralisation in services, some investigators have attributed improved outcomes to better haemostatic resuscitation (24). Increasingly in the UK, the most severely injured are treated by prehospital critical care teams who provide early specialist assessment, resuscitation and blood products to patients at the scene of injury. Since the inception of the Major Trauma Networks, there have also been developments in clinical practice which are likely to have benefited many patients with significant bleeding from liver injury, including the introduction of massive haemorrhage protocols (25, 26), increased use of tranexamic acid in bleeding trauma patients (22, 25) and earlier senior assessment on arrival to hospital, with an increase in consultant-led trauma calls (22). Additionally, the increased sensitivity of CT has led to reduced rates of missed injuries and the ability to target complications, such as bile leaks, without the need for surgery, and in turn improving outcomes (27, 28). Surgically, high grade liver trauma can be one of the most complex and challenging injuries for a trauma surgeon to manage. The presence of on-site hepatobiliary surgeons has been shown to increase odds of survival for patients following liver trauma (25). Furthermore, the adoption of damage control resuscitation (DCR), has led to improved outcomes for trauma patients, particularly those with high grade blunt liver injuries (29). With the underlying principle of DCR being reversal of the associated coagulopathy in trauma patients, achieved through effective fluid management, administration of blood products and definitive control of bleeding (30). There is also evidence to suggest that effective DCR reduces the need for intervention, with either laparotomy or angioembolisation (14).

Our study adds to the growing body of evidence that patients taken directly to an MTC with serious injuries have better outcomes. It is not possible from the current study to demonstrate the causes for these improvements, but instead we hypothesise that these improvements are due to centralisation of multidisciplinary teams that work together for trauma patient care, maturation of trauma systems that optimise patient care, and improved resuscitation techniques. Further investigations would be required to determine which factors play the greatest part in the improvements in trauma care during the establishment and growth of a Major Trauma Centre and trauma network.

Not all patients do access the MTC directly. Approximately one quarter of injured children present to local Emergency Departments as they are often brought by parents rather than ambulance (26). In all ages, analysis of more than 230,000 patients with injury severity score >15 who were reported to the Trauma, Audit and Research Network (TARN) over a three year period found that just 46% were transferred directly to an MTC and 19.3% required a secondary transfer (23). One downside of centralisation of the trauma service is the diminishing experience of surgical teams outside the MTC. Patients who arrive at their nearby hospital with a high grade liver injury may not be stable enough to be transferred, requiring the local surgeon to perform a damage control trauma laparotomy. Consideration should therefore be made to ensure access to training and updates for surgeons in trauma units to ensure skills are maintained. Additionally, with the centralisation of services, there is also a centralisation of the wider workforce who have an interest in trauma, and therefore are likely to choose to work at an MTC rather than a trauma unit. Centralisation also brings logistic challenges to patients and relatives, particularly once the acute phase of injury and recovery is passed. Patients may be relatively far from home, require long inpatient stays and multiple follow up visits. Services should consider how best to mitigate these challenges, perhaps working more closely with local units or offering virtual follow up.

As trauma systems develop and mature, the focus needs to shift towards improving functional outcomes for injured patients. Reductions in complications are a welcome move towards this, though more work is required to examine optimal recovery pathways, with consideration of psychological outcomes and focus on return to normal activity, school or work.

### 4.1. Limitations

This was an observational study, with the associated risk of bias and error that are expected with that design. Although the database was prospectively recorded, there is a risk that patients may have been missed. Even though models were designed to control for the potential demographic and injury-related confounders for the before-after comparison of outcomes, there is a risk that there were unknown or unmeasured confounding variables. We did not analyse other non-liver injuries for patients, and further investigations would be required to determine the relationships between other injuries, liver injuries and outcomes.

The study also has a relatively low number of cases, and therefore caution is advised in the interpretation of the data, and the generalisability of the findings. With the roll out of trauma centres across the entire UK taking place over a prolonged period of time, some centres may well be less-established than others, and a multicentre study may be necessary to ascertain the full picture of the management of traumatic liver injuries and associated outcomes across the UK.

### 4.2. Conclusion

In our study of 600 patients with liver trauma, patients were less likely to have complications after the establishment of the MTC, even when matched for demographic and injury variables. This was the case even though patients were older and had more comorbidities. It is likely that centralisation of key services for trauma patients leads to better overall management of patients and the reduction of trauma-related complications.

## Data Availability

The raw data supporting the conclusions of this article will be made available by the authors, without undue reservation.
